# Heterogeneous profiles of eHealth literacy in older adults with type 2 diabetes: a latent profile analysis and associated factors

**DOI:** 10.3389/fpubh.2026.1927891

**Published:** 2026-10-02

**Authors:** Zuyao Xiao, Ruiqing Cui, Jiaxin Liu, Guohua Jing, Lingyun Wei, Yiming Yang, Shuya An, Tao Liu

**Affiliations:** School of Nursing, Jinzhou Medical University, Linghe, Jinzhou, Liaoning, China

**Keywords:** type 2 diabetes mellitus, digital health, eHealth literacy, heterogeneity, latent profile analysis, patient profiles, self-management, older adults

## Abstract

**Background:**

Digital diabetes services are expanding rapidly in China, and older patients’ uptake of them depends on their eHealth literacy. This construct is usually reported as a single mean score, which conceals differences between patients sharing the same total. We aimed to identify latent profiles of eHealth literacy in Chinese older adults with type 2 diabetes and the characteristics associated with profile membership.

**Methods:**

A cross-sectional survey was conducted in the endocrinology departments of two tertiary hospitals in Jinzhou, Liaoning Province, between May and June 2026. Overall, 544 patients older adult(s) 60 years and over participated (mean age 71.4 ± 6.74 years, 52.21% female). Latent profile analysis was performed on the eight item-level scores of the Chinese eHealth Literacy Scale. Predictors for multinomial logistic regression were specified *a priori* from the Lily Model, van Dijk’s resources-and-appropriation theory and social cognitive theory, and retained regardless of univariate *p*-values. Multicollinearity was acceptable (VIF 1.10–1.69) and coefficients were checked against 1,000 bootstrap resamples.

**Results:**

A three-profile solution fitted best (entropy 0.895): Skill-Dependent (*n* = 309, 56.8%), Comprehensive Deficit (*n* = 82, 15.1%) and Comprehensive Advancement (*n* = 153, 28.1%), whose mean total scores (26.88, 17.76 and 34.72) diverged despite a sample mean of 27.71 ± 5.93. With Comprehensive Advancement as reference, older age (OR 8.50 per 10 years, 95% CI 3.45–20.94; OR 3.56, 95% CI 1.76–7.21) and greater fear of disease progression (OR 4.60 per 5 points, 95% CI 3.07–6.87; OR 1.97, 95% CI 1.46–2.67) were associated with Skill-Dependent and Comprehensive Deficit membership, respectively. Higher diabetes self-efficacy, education of high school or above, smartphone use and longer daily internet time were associated with lower odds of both lower-literacy profiles (all *p* < 0.001). Complications were associated with Skill-Dependent membership only (OR 6.85, 95% CI 2.04–22.95, *p* = 0.002).

**Conclusion:**

eHealth literacy was distributed across three qualitatively distinct profiles, so a single mean score describes this population incompletely. Age, educational attainment, device access, daily internet time, diabetes self-efficacy and fear of disease progression were associated with profile membership. Being cross-sectional, these findings describe associations rather than causal effects; profile-tailored education needs prospective evaluation.

## 1 Background

Population ageing and the rising burden of type 2 diabetes are converging global public health challenges, and this convergence is particularly pronounced in China. By 2050, China’s population older adult(s) 60 years and over is projected to reach 366 million, approximately 26% of the total population (1), and China already has the largest number of adults living with type 2 diabetes worldwide (2, 3). Because type 2 diabetes is a lifelong condition whose complications account for the majority of its clinical and economic burden (4–7), day-to-day self-management—dietary control, physical activity, medication adherence and glucose monitoring—remains the cornerstone of care in older adults (8, 9). Yet effective self-management is achieved by only a minority of patients in low- and middle-income settings, with reported rates ranging from 6.1 to 26.8% (10, 11).

Digital health services—online health education platforms, mobile applications, remote consultation and internet hospitals—have been promoted as a scalable means of closing this gap (11–14), and in China they have been written into national policy through the “Healthy China 2030” and “Internet Plus Healthcare” initiatives (15). Whether older patients actually benefit from these services depends, however, on their eHealth literacy: the ability to seek, find, understand and appraise health information from electronic sources and to apply that knowledge to a health problem (16). Evidence from several countries indicates that higher eHealth literacy is associated with better information access, more active engagement with services and more favourable diabetes-related outcomes (17, 18). International comparisons nevertheless reveal a consistent age gradient. Even in high-income settings with mature digital infrastructure, older adults report substantially lower e-HEALS scores than younger adults (19), and the gap widens in low- and middle-income countries, where device ownership, connectivity and digital-skills training are less evenly distributed (12, 20, 21). China occupies a distinctive position in this landscape: internet penetration and smartphone ownership are high at the population level, yet uptake among adults older adult(s) 60 years and over lags considerably behind, a pattern commonly described as the “grey digital divide”. For older Chinese adults with type 2 diabetes this divide has direct clinical consequences, because the digital route to self-management support is precisely the route this group is least equipped to use.

Research on eHealth literacy in this population remains dominated by variable-centred approaches—correlation and regression models that estimate the average effect of a characteristic across an entire sample (12, 22). Such models implicitly treat the population as homogeneous and therefore describe it with a single mean score. This assumption is questionable for eHealth literacy, which is explicitly multidimensional: an older patient may be able to operate a smartphone and retrieve health information (an operational skill) while being unable to judge whether that information is trustworthy (a critical appraisal skill). Two patients with identical total scores may consequently require entirely different interventions. Latent profile analysis (LPA) is a person-centred method that addresses this limitation directly, grouping individuals by their pattern of responses across indicators rather than by their overall level, and thereby recovering qualitatively distinct subgroups that a mean score conceals (23, 24). LPA has been applied productively to other constructs in nursing research (25, 26), but to our knowledge it has not been used to characterise eHealth literacy in older Chinese adults with type 2 diabetes. It is therefore not known how many distinct profiles exist in this population, what distinguishes them, or which characteristics are associated with membership of the least literate profiles—precisely the information clinicians need in order to target scarce educational resources.

Variable selection in this study was guided by three complementary frameworks. Norman and Skinner’s Lily Model conceptualises eHealth literacy as a composite of analytic literacies (traditional, media and information literacy) and context-specific literacies (computer, scientific and health literacy), and positions it as contingent on the individual’s health status, motivation and technological context (16); this framework motivated the inclusion of educational attainment, complication status and device-related variables. Van Dijk’s resources-and-appropriation theory of the digital divide distinguishes sequential forms of access—motivational, material, skills and usage—and holds that material access (device ownership) and usage access (time spent online) precede and constrain skill development (19); this informed our decision to treat smartphone use and daily internet time as distinct rather than interchangeable variables. Finally, Bandura’s social cognitive theory identifies self-efficacy as a proximal determinant of whether individuals attempt and persist with unfamiliar tasks (27), motivating the inclusion of diabetes self-efficacy, while fear of disease progression was included as its affective counterpart, which may either mobilise or impair information processing.

Accordingly, the objectives of this study were: (1) to identify latent profiles of eHealth literacy among older adults with type 2 diabetes using item-level responses to the e-HEALS; (2) to compare demographic, clinical and psychosocial characteristics—including diabetes self-efficacy and fear of disease progression—across the identified profiles; and (3) to examine which of these characteristics are associated with profile membership. The findings are intended to provide an empirical basis for profile-tailored nursing education rather than a one-size-fits-all approach.

## 2 Research methods

### 2.1 Research design

This study is a cross-sectional study conducted in accordance with the STROBE guidelines.

### 2.2 Recruitment of participants

This study employed convenience sampling. Patients were recruited from the endocrinology departments of two Grade A tertiary hospitals in Jinzhou City, Liaoning Province. Patient recruitment took place from May 2026 to June 2026. The specific inclusion and exclusion criteria are as follows.

Inclusion criteria: (1) older adult(s) 60 years or older; (2) a diagnosis of type 2 diabetes according to the American Diabetes Association Standards of Care in Diabetes (28); (3) able to comprehend the questionnaire items in Mandarin and to provide their own responses, either by reading the items independently or, where visual acuity was insufficient, by having the items read aloud verbatim by a trained researcher (see Section 2.5); and (4) willing to participate and able to provide written informed consent.

Exclusion criteria: (1) acute critical illness or severe mental illness; (2) coma, or cognitive impairment (for example Alzheimer’s disease) precluding independent communication; (3) visual or hearing impairment so severe that the participant could not comprehend the items even with the standardised assistance described in Section 2.5; and (4) a history of other serious illness.

### 2.3 Sample size calculation

Given the absence of an *a priori* formula for sample size calculation in latent profile analysis, we followed empirical recommendations. Based on Monte Carlo simulation studies, a sample size of 300–500 achieves stable model estimation when reliability is adequate (*α* > 0.80) and class separation is sufficient (29). Our study employed 8 observed indicators, yielding a sample-to-indicator ratio of 68:1 (544/8), exceeding the recommended minimum of 10–20:1 (30). To account for anticipated invalid responses (20%), we distributed 570 questionnaires. Invalid responses were identified using three pre-specified criteria: (1) completion time <180 s, (2) > 30% missing data, and (3) intra-individual inconsistency on semantically related items. Final analysis included 544 valid questionnaires.

### 2.4 Instrument

#### 2.4.1 Demographic questionnaire

For the purposes of this study, a general demographic questionnaire was developed. It included age, gender, educational attainment (high school or higher, or below high school), place of residence (urban or rural), marital status (married, unmarried, or other), daily internet usage (≤6 h per day or >6 h per day) (31), smartphone usage, presence of complications, BMI (normal, overweight, or underweight), and duration of illness.

#### 2.4.2 The Chinese version of the eHealth Literacy Scale (e-HEALS)

This scale was originally developed by Norman et al. (32) It was translated into Chinese by Guo et al. (33) The Chinese version of the eHealth Literacy Scale is divided into three dimensions: the assessment of the ability to use online health information and services (Q1–Q5), the assessment of critical thinking skills (Q6, Q7), and the assessment of decision-making skills (Q8). The scale uses a 5-point Likert scale, with each item scored from 1 to 5, resulting in a total score ranging from 8 to 40. No items are reverse-scored. A higher total score indicates higher eHealth literacy. In this study, the Cronbach’s alpha coefficient for the scale was 0.806.

#### 2.4.3 Self-Efficacy for Diabetes Scale (SED)

The Self-Efficacy for Diabetes Scale (SED) was developed by Lorig et al. (34) in the United States. It consists of nine items, which are grouped into four dimensions: diet, exercise, blood glucose control, and disease management. The scale uses a 5-point Likert scale, where a higher score indicates greater self-efficacy. The scale was adapted into Chinese by Chinese scholar Wei (35). In this study, the Cronbach’s alpha coefficient for this scale was 0.952, indicating good internal consistency.

#### 2.4.4 Fear of illness progression questionnaire (FoP-Q-SF)

This measurement tool was originally developed by Mehnert et al. (36) based on the FoP-Q scale. The scale was adapted into Chinese and introduced to China by Chinese scholar Wu Qiyun. The Chinese version of the scale consists of two subscales—physical health and social/family—comprising a total of 12 items. It uses a 5-point Likert scale, with a total score ranging from 12 to 60; a higher score indicates a greater degree of fear of disease progression among patients (37). In this study, the Cronbach’s alpha coefficient for this scale was 0.941.

### 2.5 Survey collection

Data were collected in person by nine trained researchers. All researchers completed a standardised half-day training session covering the study protocol, the informed consent procedure and a fixed script for reading items aloud and clarifying wording, in order to minimise variation between researchers. After eligibility was confirmed and written informed consent obtained, participants completed the questionnaire electronically by scanning a QR code, using either their own smartphone or a device provided by the research team.

Given the age of the participants (> = 60 years), one-to-one support was available throughout. Researchers (1) assisted with scanning the QR code, (2) read items aloud verbatim, without paraphrase, for participants whose visual acuity made independent reading difficult, and (3) clarified the meaning of unfamiliar terms using the standardised script. Researchers did not suggest, evaluate or enter answers on a participant’s behalf.

On submission, a researcher checked each questionnaire only for omitted items and for logically inconsistent responses between semantically related items. Where either was identified, the researcher drew the participant’s attention to the item concerned and invited them to review it; the decision to retain or amend the response, and the amendment itself, were made entirely by the participant. At no point did researchers judge a response to be incorrect or alter a response themselves. Questionnaires that remained incomplete or internally inconsistent after this participant-led review were retained in the dataset and handled by the pre-specified validity criteria described in Section 2.3.

### 2.6 Data analysis

#### 2.6.1 Potential profile analysis

Using Mplus 8.3, we conducted a latent profile analysis with the scores on the 8 items of the eHealth Literacy Scale as continuous observed variables. We performed a latent profile analysis of eHealth Literacy among older adult(s) patients with type 2 diabetes to identify potential heterogeneous subgroups based on their eHealth literacy proficiency levels. This study selected the 8 items of the scale (rather than the total scores of the 3 dimensions) as the observed indicators for LPA, based on the following considerations: First, analyzing each item individually allows for the identification of distinct patterns of behavioral expression within each dimension, thereby avoiding the masking effect of the total dimension scores on the individual items (38). Second, previous LPA studies in the field of nursing have already utilized item-level indicators for latent profile analysis, confirming the effectiveness of this approach in identifying more refined subgroups (25, 26). Sample Size Adequacy: This study’s sample-to-indicator ratio is 544:8 (approximately 68:1 per observed variable), which substantially exceeds the empirical guideline of 10–20 observations per indicator recommended for LPA (30). Therefore, there is adequate statistical power. Starting with a 1-class model, the number of classes was gradually increased. In accordance with guidelines in the literature, this study used the Akaike Information Criterion (AIC), the Bayesian Information Criterion (BIC), and the sample-size-adjusted BIC (aBIC) as criteria; lower values indicate a better model fit (39, 40). The Lo–Mendell–Rubin (LMR) and Bootstrap Likelihood Ratio Tests (BLRT) were used to assess whether the *k*-profile model provided a statistically significant improvement (*p* < 0.05) over the *k*-1 model. Classification accuracy was evaluated using entropy; values exceeding 0.8 indicate clear and precise profile separation (41).

#### 2.6.2 Analysis of associated factors

Categorical variables were imported into IBM SPSS 29 for further analysis. In descriptive statistical analysis, categorical variables were presented as frequencies (percentages); continuous variables were assessed for normality. Data following a normal distribution were described as mean ± standard deviation, while non-normally distributed data were described as median (interquartile range). Depending on the distribution of data across different profile groups, independent-samples t-tests, one-way ANOVA, or their nonparametric equivalents (Mann–Whitney *U* test, Kruskal-Wallis test) were used for between-group comparisons. Candidate variables for the multinomial logistic regression model were selected using a combined theory-driven and data-driven strategy. A core set of variables was specified *a priori* on the basis of the three frameworks outlined in the Introduction—age, educational attainment and complication status (Lily Model) (16), smartphone use and daily internet time (resources-and-appropriation theory of the digital divide), and diabetes self-efficacy and fear of disease progression (social cognitive theory)—and these variables were pre-specified for retention irrespective of their univariate *p*-values (27), so that theoretically relevant confounders would not be discarded on statistical grounds alone. Remaining variables were eligible for entry if they reached *p* < 0.05 in univariate analyses. In the present sample all seven pre-specified variables also satisfied this statistical criterion and no additional variable did, so the final model comprised these seven variables. Latent profile membership (three categories: Skill-Dependent, Comprehensive Deficit, Comprehensive Advancement) was entered as the dependent variable, with the ‘Comprehensive Advancement’ group designated as the reference category. Separate comparisons were conducted: (1) Skill-Dependent vs. Comprehensive Advancement, and (2) Comprehensive Deficit vs. Comprehensive Advancement. Results were reported as odds ratios (OR) with 95% confidence intervals (CI). All tests were two-sided, and a *p*-value < 0.05 was considered statistically significant. For continuous variables, odds ratios were calculated per unit increase. Prior to multivariate analysis, multicollinearity was assessed using variance inflation factors (VIF) and tolerance values for all predictor variables. VIF < 5.0 and tolerance > 0.1 were considered indicative of acceptable multicollinearity. To enhance clinical interpretability, we also calculated odds ratios for clinically meaningful increases: for the Self-Efficacy for Diabetes Scale, per 1-point increase (range 9–45) and per 10-point increase (approximately 1 SD, based on observed SD = 9.13); for the Fear of Progression Scale, per 1-point increase and per 5-point increase (approximately 0.5 SD, based on observed SD = 11.09); for age, per 1-year increase and per 10-year increase.

To verify the stability and robustness of the parameter estimates against potential sparse-data artifacts or small-subgroup constraints (e.g., in Class 2, *n* = 82), we performed an internal validation using a non-parametric bootstrap procedure with 1,000 resamples. Median regression coefficients ($\beta$) and 95% bootstrap percentile confidence intervals were calculated to confirm the empirical robustness of key digital and sociodemographic predictors.

### 2.7 Ethical considerations

This study was approved by the Scientific Research Ethics Committee of Jinzhou Medical University (approval number: JZMULL2026284) and was conducted in accordance with the principles of the Declaration of Helsinki. Before data collection, all participants were fully informed of the study’s purpose and procedures, and written informed consent was obtained from each participant. Participation was entirely voluntary, and participants were assured of their right to withdraw at any time without providing a reason. To ensure confidentiality, all personal identifiers were removed upon data collection, and the anonymized data were stored on a secure, password-protected server accessible only to the research team. The data were used solely for the academic purposes of this study; any future use of the anonymized data for related research will be subject to separate ethical approval if required.

## 3 Results

### 3.1 Demographic characteristics

After excluding invalid questionnaires, a final sample of 544 participants was included. Participants had a mean age of 71.4 ± 6.74 years, a mean EHEALTH LITERACY scale score of 27.71 ± 5.93, a mean Disease Progression Fear Scale score of 36.51 ± 11.09, and a mean Diabetes Self-Efficacy Scale score of 25.81 ± 9.13. 256 participants (47.06%) had a high school education or higher, while 288 participants (52.94%) had less than a high school education. Among them, 260 were male (47.79%) and 284 were female (52.21%). See Table 1 for detailed demographic characteristics.

**Table 1:** Demographic characteristics.

Variables	Categories	Mean (SD)	Frequency (*N*)	Percentage (%)
Gender	Female		284	52.21%
	Male		260	47.79%
Age		71.4 ± 6.74		
Place of residence	Urban		317	58.27%
	Rural		227	41.73%
Education background	High school and above		256	47.06%
	Below high school		288	52.94%
Course of the disease	<5 years		175	32.17%
	5–10 years		155	28.49%
	10 years		214	39.34%
Whether to use a smartphone	Yes		260	47.79%
	No		284	52.21%
Time spent online per day	>6 h		203	37.32%
	≤6 h		341	62.68%
Marital status	Married		329	60.48%
	Unmarried		106	19.49%
	Other		109	20.03%
Were there any complications	Yes		302	55.51%
	No		242	44.49%
BMI	Normal		237	43.57%
	Overweight		157	28.86%
	Underweight		150	27.57%
EHEALTH LITERACY Scale		27.71 ± 5.93		
Self-Efficacy for Diabetes Scale		25.81 ± 9.13		
Fear of illness progression questionnaire		36.51 ± 11.09		

Continuous variables are presented as mean ± SD; categorical variables as frequency (*N*) and percentage (%). For marital status, “Other” includes widowed, divorced, and separated participants.

### 3.2 Digital health literacy among different patient groups

In this study, scores on the eight eHealth Literacy Scale (eHEALS) items were used as indicators to fit latent profile models with one to five classes sequentially (Table 2). As the number of classes increased, AIC, BIC, and aBIC continued to decrease. However, the Lo–Mendell–Rubin (LMR) test was not significant for the 4-class model (*p* = 0.199) nor for the 5-class model (nonsignificant), whereas the 3-class model showed a significant improvement over the 2-class model (*p* < 0.01). The 3-class model also yielded an entropy of 0.895, indicating good class separation. Based on these fit indices, the 3-class solution was selected as the optimal model. Based on their item response patterns, the three classes were labeled as “Skill-Dependent” (Class 1), “Comprehensive Deficit” (Class 2), and “Comprehensive Advancement” (Class 3). The profile plot of item scores is presented in Figure 1.

**Table 2:** Model fit indices for latent profile analysis: comparison of one- to five-class models.

Model	LL	AIC	BIC	aBIC	Entropy	LMR *p*-value	BLRT *p*-value	Category probability
Class 1	−6709.7	13451.3	13520.1	13469.3				
Class 2	−6242.7	12535.4	12642.8	12563.5	0.958	*p* < 0.01	*p* < 0.01	0.16/0.84
Class 3	−6011.2	12090.4	12236.6	12128.7	0.895	*p* < 0.01	*p* < 0.01	0.57/0.15/0.28
Class 4	−5979.1	12044.1	12,229	12092.5	0.901	0.1999	*p* < 0.01	0.08/0.57/0.07/0.28
Class 5	−5949.8	12003.7	12227.2	12062.1	0.866	0.627	<0.01	0.47/0.28/0.11/0.10/0.04

**Figure 1:**
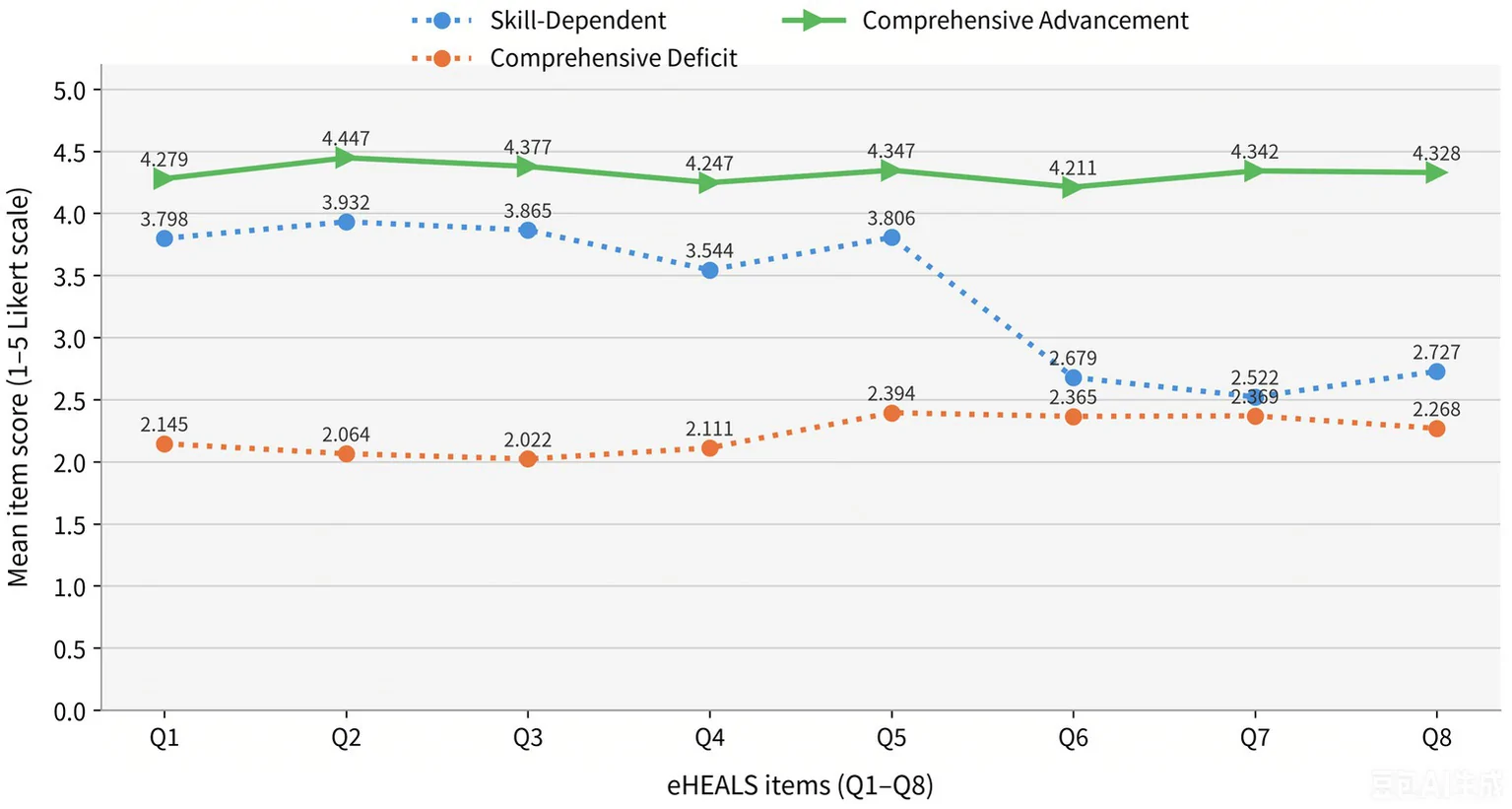
Latent profile analysis: eHealth literacy scale item scores.

#### 3.2.1 Across three profiles

The Levene test for homogeneity of variances indicated that the variances of the three dimensions and the total score of the EHEALTH LITERACY scale were heterogeneous (*p* = 0.019, 0.004, < 0.001, and 0.006, respectively). Consequently, the Games-Howell test was used for *post-hoc* pairwise comparisons. The main effects of the analysis of variance all reached the large effect size level (partial η^2^ ranged from 0.424 to 0.831). *Post-hoc* pairwise comparisons indicated that the “Comprehensive Advancement” group scored significantly higher than both the “Skill-Dependent” and “Comprehensive Deficit” groups across all dimensions and the total scale score (*p* < 0.001). Regarding the comparison between the “Skill-Dependent” and “Comprehensive Deficit” groups, the former scored significantly higher on most measures (*p* < 0.001), with the exception of the judgment ability dimension, where the difference was smaller (mean difference = 0.43, *p* = 0.029). For detailed results and effect sizes, see Table 3.

**Table 3:** Between-group differences in eHealth literacy dimensions (Welch’s ANOVA with Games-Howell *post-hoc* test).

Variables	Class 1 (*n* = 309)	Class 2 (*n* = 82)	Class 3 (*n* = 153)	Welch’s *F*	df1, df2	*p*	Partial *η*^2^
Application capability (5 items)	18.97 ± 1.78	10.76 ± 2.12	21.73 ± 1.61	1026.61	2, 198.7	<0.001	0.791
Judgment ability (2 items)	5.17 ± 1.33	4.74 ± 1.34	8.64 ± 0.99	450.94	2, 209.7	<0.001	0.625
Decision-making ability (1 item)	2.73 ± 0.99	2.26 ± 0.98	4.35 ± 0.74	199.03	2, 210.9	<0.001	0.424
eHEALS total score	26.88 ± 2.39	17.76 ± 3.00	34.72 ± 2.20	1329.72	2, 195.9	<0.001	0.831

Levene’s test indicated heterogeneity of variance for all four outcomes (*p* = 0.019, 0.004, <0.001 and 0.006, respectively); Welch’s *F* and Games-Howell *post-hoc* tests were therefore used. All pairwise *post-hoc* comparisons were significant at *p* < 0.001, with one exception: Skill-Dependent versus Comprehensive Deficit on judgment ability (mean difference = 0.43, *p* = 0.029).

### 3.3 Demographic analysis

Univariate analysis revealed that age, educational attainment, smartphone usage, daily internet usage, and the presence of comorbidities were associated with latent profile membership of eHealth literacy among older adults with type 2 diabetes. No other factors were found to influence the latent profiles of this population. See Table 4 for details.

**Table 4:** Demographic analysis.

Variables	Categories	Class 1	Class 2	Class 3	*H*/*χ*^2^	*p*
Gender	Female	165	46	73	1.912	0.384
	Male	144	36	80		
Age M (P25, P75)		74 (70, 78)	71 (66, 77)	66 (62,7 0)	125.344	<0.001
Place of residence	Urban	179	53	85	1.844	0.405
	Rural	130	29	68		
Education background	High school and above	105	38	113	65.331	<0.001
	Below high school	204	44	40		
Course of the disease	<5 years	94	29	52	3.098	0.544
	5–10 years	84	23	48		
	>10 years	131	30	53		
Whether to use a smartphone	Yes	86	45	129	132.78	<0.001
	No	223	37	24		
Time spent online per day	>6 h	48	34	121	177.395	<0.001
	≤6 h	261	48	32		
Marital status	Married	190	49	90	0.786	0.941
	Unmarried	57	18	31		
	Other	62	15	32		
Were there any complications	Yes	226	39	37	101.785	<0.001
	No	83	43	116		
BMI	Normal	127	35	75	2.877	0.580
	Overweight	91	25	41		
	Underweight	91	22	37		
Diabetes Self-Efficacy Scale (P25, P75)		20 (16,23)	29 (24,33)	36 (32,41)	320.331	<0.001
Fear of illness progression questionnaire (P25, P75)		43 (37,48)	33 (27,38)	27 (21,31)	240.105	<0.001

Categorical variables are presented as counts and compared using Pearson’s *χ*^2^ test; continuous variables are presented as median (25th, 75th percentile) and compared using the Kruskal–Wallis *H* test. This table also serves as the full cross-tabulation of every candidate predictor against profile membership; the smallest cell count is 24 and no cell is empty. M, median; SED, Self-Efficacy for Diabetes Scale; FoP-Q-SF, fear of progression questionnaire-short form.

#### 3.3.1 Assessment of multicollinearity

To ensure the statistical robustness of the latent profile analysis (LPA), we employed both the Lo–Mendell–Rubin (LMR) test and the Bootstrap Likelihood Ratio Test (BLRT) via Mplus TECH11 and TECH14 to verify the superiority of the 3-class model. Furthermore, to address potential concerns regarding model stability arising from the smaller sample size of the ‘Comprehensive Deficit’ class ($*n* = 82$), we performed a detailed cross-tabulation of all predictor variables across the identified latent profiles (see Table 4). This examination confirmed that the distribution of categorical predictors provided sufficient variation within each class, excluding the possibility that zero-cell counts or extreme data skewness biased the multinomial logistic regression estimates.

Prior to multivariate analysis, multicollinearity among predictor variables was evaluated using variance inflation factors (VIF) and tolerance values. As presented in Table 5, all VIF values ranged from 1.10 to 1.69 (well below the threshold of 5.0), and all tolerance values ranged from 0.59 to 0.91 (well above the threshold of 0.1). These results collectively indicate that the regression models are statistically stable and that multicollinearity did not pose a substantive threat to the validity of the coefficient estimates.

**Table 5:** Assessment of multicollinearity.

Variable	Tolerance	VIF
Age	0.840	1.190
Educational attainment	0.910	1.099
Smartphone use	0.801	1.249
Daily internet time	0.770	1.299
Complications	0.843	1.186
Fear of disease progression	0.701	1.426
Diabetes self-efficacy	0.593	1.686

VIF, variance inflation factor. All VIF values < 2.0 (range 1.10–1.69) and tolerance values > 0.59 (range 0.59–0.91) indicate acceptable multicollinearity, substantially below/above conventional thresholds of VIF > 5.0 and tolerance < 0.1.

### 3.4 Multiple logistic regression analysis

This study conducted a multiple logistic regression analysis using the “Comprehensive Advancement” group as the reference group.

#### 3.4.1 “Skill-Dependent” group vs. “Comprehensive Advancement” group

In multinomial logistic regression comparing ‘Skill-Dependent’ vs. ‘Comprehensive Advancement’ profiles: Older age was associated with greater odds of Skill-Dependent classification (OR = 1.239 per year, 95% CI: 1.132–1.356, *p* < 0.001; or OR = 8.50 per 10 years, 95% CI: 3.45–20.94). Higher fear of disease progression scores were associated with greater odds of Skill-Dependent classification (OR = 1.357 per point, 95% CI: 1.253–1.470, *p* < 0.001; or OR = 4.60 per 5 points, 95% CI: 3.07–6.87). Higher self-efficacy scores were associated with reduced odds of Skill-Dependent classification (OR = 0.604 per point, 95% CI: 0.541–0.675, *p* < 0.001; OR = 0.080 per 5-point increase, 95% CI: 0.046–0.140). Compared with those having <high school education, those with ≥high school education had substantially reduced odds of Skill-Dependent classification (OR = 0.047, 95% CI: 0.013–0.169, *p* < 0.001). Smartphone users compared with non-users had substantially reduced odds of Skill-Dependent classification (OR = 0.028, 95% CI: 0.008–0.102, *p* < 0.001). Those spending >6 h daily online compared with ≤6 h had substantially reduced odds of Skill-Dependent classification (OR = 0.013, 95% CI: 0.003–0.050, *p* < 0.001). Patients with complications compared with those without complications had substantially greater odds of Skill-Dependent classification (OR = 6.846, 95% CI: 2.042–22.952, *p* = 0.002) (see Table 6).

**Table 6:** Multivariate logistic regression: “Skill-Dependent” vs. “Comprehensive Advancement” profile membership.

Variables	*B* (SE)	Wald	*p*	OR (95% CI) per unit	OR (95% CI) per clinically meaningful increase
Age (years)	0.214 (0.046)	21.776	<0.001	1.239 (1.132–1.356) per year	*per 10 years*: 8.50 (3.45–20.94)
Fear of progression (FoP-Q-SF)	0.305 (0.041)	56.165	<0.001	1.357 (1.253–1.470) per point	*per 5 points*: 4.60 (3.07–6.87)
Diabetes self-efficacy (SED)	−0.504 (0.056)	79.881	<0.001	0.604 (0.541–0.675) per point	*per 5 points*: 0.080 (0.046–0.140)
Education (≥High school vs. <)	−3.055 (0.652)	21.971	<0.001	0.047 (0.013–0.169)	—
Smartphone use (Yes vs. No)	−3.562 (0.653)	29.719	<0.001	0.028 (0.008–0.102)	—
Daily internet time (>6 h vs. ≤6 h)	−4.333 (0.683)	40.237	<0.001	0.013 (0.003–0.050)	—
Complications (Yes vs. No)	1.924 (0.617)	9.712	0.002	6.846 (2.042–22.952)	—

The reference category is “Comprehensive Advancement.” OR, odds ratio; CI, confidence interval. Clinically meaningful increases were chosen as: age (10 years, ~1.5 SD), self-efficacy (5 points, ~0.5 SD), and fear of progression (5 points, ~0.5 SD). This ensures symmetric and clinically intuitive interpretation across continuous predictors; a 5-point change is more readily achievable in clinical practice than a 10-point change for the SED (scale range 9–45). For reference, the per-1-point OR for self-efficacy in this comparison was 0.604 (95% CI: 0.541–0.675).

#### 3.4.2 “Comprehensive Deficit” group vs. “Comprehensive Advancement” group

Age and fear of disease progression were both significantly associated with ‘Comprehensive Deficit’ classification. Specifically, each 1-year increment in age was associated with 13.5% higher odds of Comprehensive Deficit classification (OR = 1.135, 95% CI: 1.059–1.217, *p* < 0.001), and each 1-point increment in fear of progression score with 14.5% higher odds (OR = 1.145, 95% CI: 1.078–1.217, *p* < 0.001). High school education or higher (OR = 0.132), smartphone use (OR = 0.112), and daily internet use exceeding 6 h (OR = 0.110) were each associated with lower odds of Comprehensive Deficit membership (all *p* < 0.001). The presence or absence of complications was not statistically significant in this comparison (OR = 2.061, *p* = 0.133). “Higher self-efficacy scores were associated with reduced odds of comprehensive deficit classification (OR = 0.813 per point, 95% CI: 0.754–0.877, *p* < 0.001; OR = 0.355 per 5-point increase, 95% CI: 0.244–0.519). See Table 7 for details.

**Table 7:** Multivariate logistic regression: “Comprehensive Deficit” vs. “Comprehensive Advancement” profile membership.

Variables	*B* (SE)	Wald	*p*	OR (95% CI) per unit	OR (95% CI) per clinically meaningful increase
Age (years)	0.127 (0.036)	12.727	<0.001	1.135 (1.059–1.217) per year	*per 10 years*: 3.56 (1.76–7.21)
Fear of progression (FoP-Q-SF)	0.136 (0.031)	19.123	<0.001	1.145 (1.078–1.217) per point	*per 5 points*: 1.97 (1.46–2.67)
Diabetes self-efficacy (SED)	−0.207 (0.038)	28.993	<0.001	0.813 (0.754–0.877) per point	*per 5 points*: 0.355 (0.244–0.519)
Education (≥High school vs. <)	−2.027 (0.522)	15.106	<0.001	0.132 (0.047–0.366)	—
Smartphone use (Yes vs. No)	−2.191 (0.525)	17.403	<0.001	0.112 (0.040–0.313)	—
Daily internet time (>6 h vs. ≤6 h)	−2.210 (0.500)	19.538	<0.001	0.110 (0.041–0.292)	—
Complications (Yes vs. No)	0.723 (0.481)	2.260	0.133	2.061 (0.803–5.292)	—

The reference category is “Comprehensive Advancement.” OR, odds ratio; CI, confidence interval. Clinically meaningful increases are defined symmetrically as in Table 6: age (10 years), self-efficacy (5 points), and fear of progression (5 points). All per-unit estimates are retained for statistical completeness; the clinically meaningful columns are provided to facilitate practical interpretation.

#### 3.4.3 Robustness and internal validation via bootstrap resampling

To address potential estimation bias for variables with pronounced odds ratios (e.g., smartphone usage, daily internet time, and diabetes complications), an internal validation was performed using non-parametric bootstrap resampling with 1,000 iterations (all iterations converged successfully). As presented in Table 8, the bootstrap median coefficients aligned closely with the primary model point estimates: smartphone use (median *β* = −3.774, 95% CI: −5.514 to −2.471 for Class 1 vs. Class 3; median *β* = −2.343, 95% CI: −3.800 to −1.148 for Class 2 vs. Class 3), daily internet time > 6 h (median *β* = −4.624, 95% CI: −6.349 to −3.409 for Class 1 vs. Class 3; median *β* = −2.370, 95% CI: −3.690 to −1.413 for Class 2 vs. Class 3), educational attainment (*β* = −3.290, 95% CI: −4.921 to −2.012), and diabetes complications (*β* = 2.030, 95% CI: 0.762 to 3.422). Notably, all 95% bootstrap percentile confidence intervals excluded zero, demonstrating that the associations identified in the primary multinomial logistic regression were statistically robust and not artifacts of data sparseness or model instability.

**Table 8:** Robustness analysis: nonparametric bootstrap validation (1,000 resamples) for multinomial logistic regression estimates.

Comparison	Variable	Point estimate (B)	Bootstrap median (B)	Bootstrap 95% CI	Bias (point—median)
C1 vs C3	Smartphone use (Yes vs. No)	−3.562	−3.774	−5.514 to −2.471	0.212
	Daily internet >6 h (vs. ≤6 h)	−4.333	−4.624	−6.349 to −3.409	0.291
	Education (≥High school vs. <)	−3.055	−3.290	−4.921 to −2.012	0.235
	Complications (Yes vs. No)	1.924	2.030	0.762 to 3.422	−0.106
C2 vs C3	Smartphone use (Yes vs. No)	−2.191	−2.343	−3.800 to −1.148	0.152
	Daily internet >6 h (vs. ≤6 h)	−2.210	−2.370	−3.690 to −1.413	0.160

C1 = Skill-Dependent; C2 = Comprehensive Deficit; C3 = Comprehensive Advancement. CI, confidence interval. Bias represents the difference between the point estimate from the original sample and the median of the bootstrap distribution. All models converged successfully across 1,000 resamples.

## 4 Discussion

To our knowledge, this is the first study to use latent profile analysis to characterize eHealth literacy heterogeneity among Chinese older adults with type 2 diabetes. The results indicate that eHealth literacy in this population is not homogeneously distributed but rather manifests in three distinct latent profiles: ‘Skill-Dependent,’ ‘Comprehensive Deficit,’ and ‘Comprehensive Advancement.’ Although the mean total score on the EHEALTH LITERACY scale was 27.71 ± 5.93, indicating an overall moderate level, “Skill-Dependent” patients scored poorly on the evaluation and decision-making dimensions, while “Comprehensive Deficit” patients scored significantly lower across all three dimensions; together, these two subgroups accounted for over 50% of the sample. This finding overcomes the limitations of previous approaches that relied solely on mean scores to describe EHEALTH LITERACY levels. It profoundly reveals the internal heterogeneity among older adult(s) patients with type 2 diabetes, suggesting that future nursing practices should not adopt a “one-size-fits-all” strategy when improving this group’s eHealth literacy, but rather implement targeted education tailored to different patient categories.

### 4.1 Individual-level factors associated with eHealth literacy profiles

#### 4.1.1 Age and eHealth literacy profile membership

Older age and lower educational attainment were significantly associated with membership in the Skill-Dependent and Comprehensive Deficit profiles. While our cross-sectional study did not directly assess neurocognitive or sensory functions, previous literature suggests that age-related changes in fluid intelligence, executive processing, and visual–auditory acuity may represent potential physiological barriers that impede the rapid adoption of digital technologies (17, 42, 43). However, these unmeasured factors should be treated as plausible hypotheses rather than confirmed causal pathways in the current sample. Regarding education, older adults with higher educational attainment often possess richer baseline cognitive resources and broader health-seeking experiences, which may facilitate information evaluation (44, 45). Rather than reflecting occupational stereotypes, this educational disparity underscores the need for simplified, multimodal (audio-visual) educational materials to support older adults with limited formal schooling (46).

#### 4.1.2 Educational attainment and profile membership

Educational attainment further distinguished the Comprehensive Advancement profile from the Skill-Dependent and Comprehensive Deficit profiles, a pattern consistent with Van Dijk’s digital divide theory (19, 44, 47). The reasons for this phenomenon can be understood from the following perspectives. First, patients with higher levels of education possess better abilities to gather and process information, and these abilities are highly correlated with the components of eHealth literacy—namely, information retrieval, application, and critical thinking (45). Second, cohort differences in the timing of first exposure to digital technology mean that older adults with more formal schooling have generally had longer and more varied opportunities to acquire digital skills. This is best read as a difference in accumulated opportunity rather than as a characteristic of any particular occupational group. In summary, when working to improve the eHealth literacy of older adults with diabetes in the future, special attention should be given to those with lower levels of education. Easy-to-understand multimedia materials (such as text and images, audio, and short videos) should be used in place of plain text to bridge the educational gap (46).

### 4.2 The relationship between smartphone use, daily internet usage, and eHealth literacy

Smartphone ownership and daily internet usage time distinguished the Comprehensive Advancement profile from the other two groups. In accordance with Van Dijk’s digital divide theory (19, 47, 48), physical access to a smartphone represents the foundational threshold for engaging with digital health platforms. However, our findings should not be construed as recommending excessive daily screen time (e.g., >6 h) for older adults. Prolonged internet use does not inherently equate to constructive health information seeking and may even contribute to digital fatigue or exposure to misinformation. Interventions should prioritize the quality and critical appraisal of online health navigation rather than merely increasing total screen hours (49, 50).

### 4.3 Disease-related and psychosocial factors

#### 4.3.1 Diabetes complications and motivation-function trade-offs

The presence of diabetes complications was significantly associated with a higher likelihood of membership in the Skill-Dependent profile compared to the Comprehensive Advancement profile, whereas no significant association was observed for the Comprehensive Deficit group. Several mechanisms may explain this differential pattern. First, complications necessitate more frequent clinical encounters, which may stimulate basic information-seeking behaviors and operational device use (51). However, specific physical limitations (e.g., retinopathy or neuropathy) combined with heightened disease burden may create barriers to higher-level critical appraisal and decision-making. Conversely, for the Comprehensive Deficit profile, foundational barriers—such as advanced age and non-ownership of digital devices—supersede the impact of complication status (52). Interventions for Skill-Dependent patients with complications should therefore integrate age-friendly accessibility design with critical evaluation training (53).

#### 4.3.2 Fear of disease progression and cognitive load

Higher fear of disease progression was associated with increased odds of classification into both the Skill-Dependent and Comprehensive Deficit profiles relative to Comprehensive Advancement, with a more pronounced association observed for the Skill-Dependent profile. While moderate health concerns can prompt proactive information search, excessive anxiety may induce psychological distress that impairs working memory, attention, and critical information processing (51). Consequently, for older adults experiencing high health-related anxiety, simply encouraging more internet use is insufficient. Priority should be given to psychosocial counseling, cognitive reappraisal based on cognitive behavioral therapy (CBT) principles (54), or mindfulness-based stress reduction (55) to alleviate emotional burden before delivering digital literacy training.

#### 4.3.3 Self-efficacy and profile membership

Diabetes self-efficacy was strongly associated with eHealth literacy profile membership: higher self-efficacy markedly increased the likelihood of belonging to the Comprehensive Advancement profile rather than the Skill-Dependent or Comprehensive Deficit profiles. In accordance with Social Cognitive Theory (27), self-efficacy serves as a proximal internal driver of health behaviors (56). Patients with high self-efficacy actively explore digital platforms, critically cross-examine online health information, and apply recommendations to their daily routine (56, 57). In contrast, patients with low self-efficacy often adopt avoidance patterns and harbor negative expectations regarding digital tools (57). Nursing interventions should focus on bolstering self-efficacy through supportive education, vicarious experiences from peers, and structured reciprocal support to foster digital confidence and active self-management (58).

### 4.4 Limitations

This study has several limitations that warrant consideration. First, participants were recruited through convenience sampling solely from the endocrinology departments of two tertiary hospitals in Liaoning Province, which may limit the generalizability of the findings to community-dwelling populations and less-developed rural areas; future cross-regional, multicenter studies are warranted. Second, the cross-sectional design precludes causal inferences regarding the temporal relationships between predictors and eHealth literacy profiles. Third, reliance on self-reported questionnaires may introduce recall and social desirability bias, and future studies should incorporate objective behavioral measures, such as device usage logs or digital skill performance tests. Fourth, although the relatively small proportion of Class 2 (*n* = 82, 15.1%) resulted in sparse cell distributions for certain binary behavioral indicators (such as smartphone non-use and prolonged internet time), our 1,000-resample bootstrap validation confirmed that all core predictor estimates remained statistically stable, with median parameters closely matching original estimates and all 95% confidence intervals entirely excluding zero (Table 8). Nonetheless, replication in larger, multicenter cohorts is warranted to obtain even more precise point estimates. Finally, we assessed diabetes self-efficacy rather than actual self-management behaviors (e.g., diet, exercise, and blood glucose monitoring), and future research should directly link eHealth literacy profiles with validated measures of self-management behavior.

## 5 Conclusion

This study conducted the first latent profile analysis of eHealth literacy among older adult(s) Chinese patients with type 2 diabetes, revealing internal heterogeneity and identifying three latent categories: “Skill-Dependent,” “Comprehensive Deficit,” and “Comprehensive Advancement.” These findings overcome the limitations of traditional approaches that simply classify patients’ eHealth literacy as “moderate” based on a mean score (27.71 ± 5.93). Analysis indicates that age, educational level, smartphone usage, daily internet usage duration, self-efficacy, and fear of disease progression differentiated the identified subgroups different subgroups. Based on these findings, future healthcare providers should implement tailored interventions for different patient subgroups: for “Skill-Dependent” patients, the focus should be on enhancing critical thinking and cognitive abilities to guide their transition toward the “Comprehensive Advancement” subgroup; for “Comprehensive Deficit” patients, efforts should focus on improving their acceptance of electronic devices to gradually enhance their eHealth literacy; and for “Comprehensive Advancement” patients, they should be engaged as peer educators or health ambassadors to support progression of the other two subgroups toward higher eHealth literacy levels.

## Data Availability

The original contributions presented in the study are included in the article/supplementary material, further inquiries can be directed to the corresponding author.

## References

[1] Lobanov-Rostovsky S He Q Chen Y Liu Y Wu Y Liu Y . Growing old in China in socioeconomic and epidemiological context: systematic review of social care policy for older people. BMC Public Health. (2023) 23:1272. doi: 10.1186/s12889-023-15583-1, 37391766 PMC10311713

[2] Ong KL Stafford LK McLaughlin SA Boyko EJ Vollset SE Smith AE . Global, regional, and national burden of diabetes from 1990 to 2021, with projections of prevalence to 2050: a systematic analysis for the global burden of disease study 2021. Lancet. (1990) 402:203–34. doi: 10.1016/s0140-6736(23)01301-6, 37356446 PMC10364581

[3] Sun H Saeedi P Karuranga S Pinkepank M Ogurtsova K Duncan BB . IDF diabetes atlas: global, regional and country-level diabetes prevalence estimates for 2021 and projections for 2045. Diabetes Res Clin Pract. (2022) 183:109119. doi: 10.1016/j.diabres.2021.109119, 34879977 PMC11057359

[4] Petrović N Milosavljević M Gojak R Đešević M Lakić D Stević I . Costs of treating type 2 diabetes mellitus and its complications. Biotechnol Biotechnol Equip. (2024) 38:2399941. doi: 10.1080/13102818.2024.2399941

[5] Burkart K Causey K Cohen AJ Wozniak SS Salvi DD Abbafati C . Estimates, trends, and drivers of the global burden of type 2 diabetes attributable to PM2· 5 air pollution, 1990–2019: an analysis of data from the global burden of disease study 2019. Lancet Planet Health. (2022) 6:e586–600. 10.1016/S2542-5196(22)00122-X PMC9278144 35809588

[6] Tomic D Shaw JE Magliano DJ. The burden and risks of emerging complications of diabetes mellitus. Nat Rev Endocrinol (2022) 2022;18:525–539, doi: 10.1038/s41574-022-00690-7, 35668219. PMC9169030

[7] Hidayat B Ramadani RV Rudijanto A Soewondo P Suastika K Ng JYS. Direct medical cost of type 2 diabetes mellitus and its associated complications in Indonesia. Value Health Reg Issues. (2022) 28:82–9. 10.1016/j.vhri.2021.04.006 34839111

[8] Zhang A Wang J Wan X Guo Z Zhang Z Zhao S . The mediating effect of self-efficacy on the relationship between diabetes self-management ability and patient activation in older adults with type 2 diabetes. Geriatr Nurs. (2023) 51:136–42. doi: 10.1016/j.gerinurse.2023.02.017, 36940508

[9] LeRoith D Biessels GJ Braithwaite SS Casanueva FF Draznin B Halter JB . Treatment of diabetes in older adults: an endocrine society clinical practice guideline. J Clin Endocrinol Metab. (2019) 104:1520–74. doi: 10.1210/jc.2019-00198, 30903688 PMC7271968

[10] Banu B Khan MMH Ali L Barnighausen T Sauerborn R Souares A. Pattern and predictors of non-adherence to diabetes self-management recommendations among patients in peripheral district of Bangladesh. Trop Med Int Health (2024) 2024;29:233–242, doi: 10.1111/tmi.13966, 38221661.

[11] Lin M Chen T Fan G. Current status and influential factors associated with adherence to self-monitoring of blood glucose with type 2 diabetes mellitus patients in grassroots communities: a cross-sectional survey based on information-motivation-behavior skills model in China. Front Endocrinol (Lausanne). (2023) 14:1111565. doi: 10.3389/fendo.2023.1111565, 37441499 PMC10335788

[12] Cui J Wang D Hu Y Yao Y Wang X Huang X . eHealth literacy mediates self-advocacy and self-management in older adults with diabetes. BMC Geriatr. (2026) 26:54. doi: 10.1186/s12877-025-06859-9, 41382291 PMC12801930

[13] Shan R Sarkar S Martin SS. Digital health technology and mobile devices for the management of diabetes mellitus: state of the art. Diabetologia. (2019) 62:877–87. doi: 10.1007/s00125-019-4864-7, 30963188

[14] Beck J Greenwood DA Blanton L Bollinger ST Butcher MK Condon JE . 2017 national standards for diabetes self-management education and support. Diabetes Educ. (2018) 44:35–50. doi: 10.1177/0145721718754797, 29346744

[15] Yang F Shu H Zhang X. Understanding “internet plus healthcare” in China: policy text analysis. J Med Internet Res. (2021) 23:e23779. doi: 10.2196/23779, 34309581 PMC8367124

[16] Norman CD Skinner HA. eHealth literacy: essential skills for consumer health in a networked world. J Med Internet Res. (2006) 8. doi: 10.2196/jmir.8.2.e9, 16867972 PMC1550701

[17] Khedmat L Faeghi A Raei M Naimi E Rahmati F. The relationship between health literacy and health-promoting behaviors in patients with type 2 diabetes mellitus: evidence from a military hospital-based study in Iran. J Health Literacy. (2026) 11:9–22. doi: 10.22038/jhl.2025.85614.1706

[18] Stephen DA Nordin A Johansson UB Nilsson J. eHealth literacy and its association with demographic factors, disease-specific factors, and well-being among adults with type 1 diabetes: cross-sectional survey study. JMIR Diabetes. (2025) 10:e66117. doi: 10.2196/66117, 40163628 PMC11975121

[19] Van Dijk JA. The Deepening Divide: Inequality in the Information Society. SAGE Publications, Inc. (2005).

[20] Shekofteh M Ghaedi R Valizadeh-Haghi S Baghestani A. E-health literacy among healthcare students: the effect of demographic variables. J Health Literacy. (2022) 7:37–45. doi: 10.22038/jhl.2022.62808.1257

[21] Li S Cui G Yin Y Wang S Liu X Chen L. Health-promoting behaviors mediate the relationship between eHealth literacy and health-related quality of life among Chinese older adults: a cross-sectional study. Qual Life Res. (2021) 30:2235–43. doi: 10.1007/s11136-021-02797-2, 33661455 PMC8298362

[22] Cui J Hu Y Yao Y Huang X Wang X. eHealth literacy, self-management, and selected clinical and social correlates of nutrition-inflammation level among older adults with diabetes. BMC Health Serv Res. (2026) 26. doi: 10.1186/s12913-026-14726-5, 42129807 PMC13343649

[23] Wang X Tu Q Huang D Jin P Cai X Zhao H . A latent profile analysis of emotional expression among patients with unintentional injuries. BMC Psychiatry. (2022) 22:729. doi: 10.1186/s12888-022-04390-4, 36424565 PMC9694566

[24] Hensel DJ. Using latent profile analysis and related approaches in adolescent health research. J Adolesc Health. (2020) 67:153–4. doi: 10.1016/j.jadohealth.2020.05.005, 32739021

[25] Zhang Y Tian L Zhang Y Li Y Jiang H. Patient safety competency among newly recruited nurses in China: a latent profile analysis. BMC Nurs. (2026) 25. doi: 10.1186/s12912-026-04494-2, 41749215 PMC13112748

[26] Gonzalez O MacKinnon DP Muniz FB. Extrinsic convergent validity evidence to prevent jingle and jangle fallacies. Multivariate Behav Res. (2021) 56:3–19. doi: 10.1080/00273171.2019.1707061, 31958017 PMC7369230

[27] Bandura A. Self-efficacy: toward a unifying theory of behavioral change. Psychol Rev. (1977) 84:191–215. doi: 10.1037/0033-295X.84.2.191, 847061

[28] American DAPP. 2. Diagnosis and classification of diabetes: standards of care in diabetes—2024. Diabetes Care. (2023) 47:S20. 10.2337/dc24-S002 PMC10725812 38078589

[29] Nylund KL Asparouhov T Muth ENBO. Deciding on the number of classes in latent class analysis and growth mixture modeling: a Monte Carlo simulation study. Struct Equ Model Multidiscip J. (2007) 14:535–69.

[30] Nylund-Gibson K Choi AY. Ten frequently asked questions about latent class analysis. Transl Issues Psychol Sci. (2018) 4:440–61. doi: 10.1037/tps0000176

[31] Jingjie W Yang L Jing Y Ran L Yiqing X Zhou N. Sedentary time and its association with risk of cardiovascular diseases in adults: an updated systematic review and meta-analysis of observational studies. BMC Public Health. (2022) 22:286. doi: 10.1186/s12889-022-12728-6, 35148747 PMC8840786

[32] Norman CD Skinner HA. EHEALS: the eHealth literacy scale. J Med Internet Res. (2006) 8:e507. doi: 10.2196/jmir.8.4.e27, 17213046 PMC1794004

[33] Guo SJ. Exploration on the sinicization and applicability of the eHEALS health literacy scale. Zhongguo Jian Kang Jiao Yu. (2013) 29:106–8.

[34] Lorig K. Outcome Measures for Health Education and Other Health Care Interventions. Sage (1996).

[35] Wei J. Study on the Correlation among Knowledge, Self-Efficacy and Self-Management Behavior in Elderly Diabetic Patients in Rural Areas. Hangzhou Normal University (2013).

[36] Mehnert A Herschbach P Berg P Henrich G Koch U. Progredienzangst bei Brustkrebspatientinnen - Validierung der Kurzform des Progredienzangstfragebogens PA-F-KF/fear of progression in breast cancer patients – validation of the short form of the fear of progression questionnaire (FoP-Q-SF). Z Psychosom Med Psychother. (2006) 52:274–88. doi: 10.13109/zptm.2006.52.3.274, 17156600

[37] Wu QY. Sinicization and reliability and validity analysis of the fear of progression questionnaire-short form in cancer patients. Chin J Nurs. (2020) 55:1200–4.

[38] Marsh HW Lüdtke O Trautwein U Morin AJ. Classical latent profile analysis of academic self-concept dimensions: synergy of person-and variable-centered approaches to theoretical models of self-concept. Struct Equ Model Multidiscip J. (2009) 16:191–225. doi: 10.1080/10705510902751010

[39] Băjenaru L Balog A Dobre C Drăghici R Prada GI. Latent profile analysis for quality of life in older patients. BMC Geriatr. (2022) 22:848 10.1186/s12877-022-03518-1 PMC9652949 36368920

[40] Ferguson SL Moore EW Hull DM. Finding latent groups in observed data: a primer on latent profile analysis in Mplus for applied researchers. Int J Behav Dev. (2020) 44:458–68. doi: 10.1177/0165025419881721

[41] Guo Y Zhou M Yan X Liu Y Wang L. A latent profile analysis of heterogeneity in self-management behavior of gestational diabetes mellitus patients. BMC Pregnancy Childbirth. (2025) 25:159. doi: 10.1186/s12884-025-07292-x, 39953435 PMC11827252

[42] Dixon E Anderson J Lazar A. Understanding how sensory changes experienced by individuals with a range of age-related cognitive changes can affect technology use. ACM Trans Accessible Comput (TACCESS). (2022) 15:1–33. doi: 10.1145/3511906, 35919105 PMC9340800

[43] Salthouse T. Consequences of age-related cognitive declines. Annu Rev Psychol. (2012) 63:201–26. doi: 10.1146/annurev-psych-120710-100328, 21740223 PMC3632788

[44] Choi NG DiNitto DM. The digital divide among low-income homebound older adults: internet use patterns, eHealth literacy, and attitudes toward computer/internet use. J Med Internet Res. (2013) 15:e93. doi: 10.2196/jmir.2645, 23639979 PMC3650931

[45] Neter E Brainin E. eHealth literacy: extending the digital divide to the realm of health information. J Med Internet Res. (2012) 14:e19. doi: 10.2196/jmir.1619, 22357448 PMC3374546

[46] Kim H Xie B. Health literacy in the eHealth era: a systematic review of the literature. Patient Educ Couns. (2017) 100:1073–82. doi: 10.1016/j.pec.2017.01.015, 28174067

[47] Richtering SS Hyun K Neubeck L Coorey G Chalmers J Usherwood T . eHealth literacy: predictors in a population with moderate-to-high cardiovascular risk. JMIR Hum Factors. (2017) 4:e4. doi: 10.2196/humanfactors.6217, 28130203 PMC5303199

[48] Shi Y Ma D Zhang J Chen B. In the digital age: a systematic literature review of the e-health literacy and influencing factors among Chinese older adults. J Public Health (Oxf). (2023) 31:679–87. doi: 10.1007/s10389-021-01604-z, 34104627 PMC8175232

[49] Wildenbos GA Peute L Jaspers M. Aging barriers influencing mobile health usability for older adults: a literature based framework (MOLD-US). Int J Med Inform. (2018) 114:66–75. doi: 10.1016/j.ijmedinf.2018.03.012, 29673606

[50] Vicente MIAR Madden G. Assessing eHealth skills across Europeans. Health Policy Technol. (2017) 6:161–8. doi: 10.1016/j.hlpt.2017.04.001

[51] Mullainathan S Shafir E Shafir E. Scarcity: Why Having too Little means so much. (2013)

[52] Van Deursen AJ Van Dijk JA. The first-level digital divide shifts from inequalities in physical access to inequalities in material access. New Media Soc. (2019) 21:354–75. doi: 10.1177/1461444818797082, 30886536 PMC6380454

[53] Diviani N Van Den Putte B Giani S van Weert JC. Low health literacy and evaluation of online health information: a systematic review of the literature. J Med Internet Res. (2015) 17:e112. doi: 10.2196/jmir.4018, 25953147 PMC4468598

[54] Gonzalez JS Tanenbaum ML Commissariat PV. Psychosocial factors in medication adherence and diabetes self-management: implications for research and practice. Am Psychol. (2016) 71:539–51. doi: 10.1037/a0040388, 27690483 PMC5792162

[55] Whitebird RR Kreitzer MJ Vazquez-Benitez G Enstad CJ. Reducing diabetes distress and improving self-management with mindfulness. Soc Work Health Care. (2018) 57:48–65. doi: 10.1080/00981389.2017.1388898, 29064772 PMC9083374

[56] Cajita MI Hodgson NA Budhathoki C Han HER. Intention to use mHealth in older adults with heart failure. J Cardiovasc Nurs. (2017) 32:E1–7. doi: 10.1097/JCN.0000000000000401, 28248747 PMC5573659

[57] Zhao YC Zhao M Song S. Online health information seeking behaviors among older adults: systematic scoping review. J Med Internet Res. (2022) 24:e34790. doi: 10.2196/34790, 35171099 PMC8892316

[58] Heisler M Vijan S Makki F Piette JD. Diabetes control with reciprocal peer support versus nurse care management: a randomized trial. Ann Intern Med. (2010) 153:507–15. doi: 10.7326/0003-4819-153-8-201010190-00007, 20956707 PMC4117390

